# Subjective Appraisal and Orientations in Difficult Life Situations as Predictors of Coping Strategies

**DOI:** 10.11621/pir.2021.0312

**Published:** 2021-09-30

**Authors:** Ekaterina V. Bityutskaya, Aleksey A. Korneev

**Affiliations:** Faculty of psychology, Lomonosov Moscow State University, Moscow, Russia

**Keywords:** Cognitive appraisal, ways of coping, planful problem-solving, positive reappraisal, wishful thinking, self-blame, approach coping, avoidance coping, life situation

## Abstract

**Background:**

Many studies have shown that problem-focused coping and a positive reappraisal of one’s situation are the most conducive to achieving life goals and psychological well-being, whereas avoidance coping and self-blame have a negative impact on well-being. But there is not enough data on what the predictors of these coping strategies are in the situational context.

**Objective:**

To assess the combined influence of subjective appraisal (uncontrollability, unclearness, negative emotions) and orientations in difficult situations (by drive and rejection) on planful problem solving, positive reappraisal, wishful thinking (fantasizing), and self-blame.

**Design:**

The research has a survey design. The sample consisted of 637 adult participants who analyzed difficult situations in their lives associated with achieving significant life goals of various types (N = 637; 60% female; M_age_ = 24.2; SD = 6.25). Two alternative structural models were assessed, which include subjective appraisals of the situation (uncontrollability, unclearness, intensity of negative emotions), orientations in difficult situations (drive and rejection), and ways of coping (planful problem-solving, positive reappraisal, wishful thinking, and self-blame).

**Results:**

The first model, in which all cognitive appraisals and orientations in difficult situations directly influence coping strategies, has relatively low fit indices. The second model, in which the influence of cognitive appraisal was partially mediated by orientations in difficult situations, has better fit indices. In life situations involving solution of a difficult task, the strongest predictor of problem-focused coping and positive reappraisal is the “drive” orientation of being attracted to difficulties, which mediates the influence of subjective control and emotions on these ways of coping. An orientation away from difficulties, “rejection,” mediates the influence of unclearness and negative emotions on fantasizing and self-blame. A low level of subjective control directly affects self-blame and the avoidance of problem-solving. Negative emotions are a weak predictor of self-blame.

**Conclusion:**

Interaction between the subject and the situation involves appraisal of difficulty, which influences orientation in difficult situations. In turn, orientations are predictors of coping strategies. The characteristics of the psychological situation determine coping, which may be oriented toward approach to or avoidance of the goal.

## Introduction

The rapidly growing body of data on the psychology of coping indicates that coping strategies are a significant factor of psychological well-being. These studies show that predictors of life satisfaction, happiness, and physical and mental health include planning, proactive coping, positive reappraisal, self-blame, and support of significant others (Bakracheva, 2019; Groth et al., 2019; Panahi, 2016; Park & Adler, 2003; Park et al., 2020). However, the question arises of the determinants of the coping strategies. In this article, we analyze cognitive predictors of coping strategies (namely, perceived situational characteristics) in situations of life goal attainment.

The article is aimed first of all at broadening the research field of perceived components of a situation. Second, it may broaden psychological activity theory (Leontiev, 1975; Sokolova, 2021), by adding empirical data on coping with difficult life situations.

### Ways of Coping and Their Cognitive Predictors

According to the transactional theory of stress and coping, coping is understood as “constantly changing cognitive and behavioral efforts to manage specific external and/or internal demands that are appraised as taxing or exceeding the resources of the person” (Lazarus & Folkman, 1984, p. 141).

Many studies have shown the influence of cognitive appraisal on coping. Cognitive appraisal, following the transactional model of stress and coping, is seen as “the process of categorizing an encounter, and its various facets, with respect to its significance for well-being” of the subject, as a factor of coping dynamics (Lazarus & Folkman, 1984, p. 31). Multiple studies demonstrate the variability of coping, depending on whether stress is perceived as a loss, a threat, or a challenge (Aldwin, 2011; Folkman & Moskowitz, 2004).

The concept of meaning-making distinguishes between situational and global meaning. Global meaning refers to people’s life goals, beliefs, and expectations about the world (Park & Folkman, 1997). A number of studies have been based on the proposition that stress is experienced when the meaning a person attaches to an event is incompatible with that person’s global meanings (Geninet & Marchand, 2007; Park & George, 2018).

It should be noted that identification of cognitive predictors of coping is connected with the problem of the differences between some coping methods and cognitive appraisal, a problem which has been a subject of debate since the early 1990s, but has yet to find an unambiguous answer (Schwarzer & Schwarzer, 1996).

We believe the problem can be resolved if the subject’s goal is seen as part of the coping process. R. Lazarus, in developing the main principles of his theory, stressed the importance of the very fact that an attempt to cope was made, regardless of whether it was successful (Lazarus, 1991). According to more recent interpretations, coping can be understood as “positive coping,” i.e., the creation of resources that contribute to the achievement of complex goals and personal growth (Schwarzer & Knoll, 2003). This approach makes goal achievement an important criterion of successful coping. In our case, this involves the identification of special types of situations that require an effort, if a significant, difficult task is to be accomplished.

### Coping Functions in Solving Life Tasks

In the contemporary view, “tasks” are situations that call for activity of the subject, the performance of some actions to solve the task and focus on the future (i.e., not just on the current situation, but also on the solution of future tasks) (Rauthmann & Sherman, 2018). Such situations call for coping as a process that makes it possible “to adapt to these changing opportunities in order to experience well-being” (Cantor & Sanderson, 1999, p. 230). Coping performs two functions in the solution of difficult tasks. First, it provides an *approach* to the goal through planful efforts. Second, in working toward a goal, the person needs to resist distracting influences, because realization of intentions may be complicated by attempts to *avoid* the difficulty (Brandstädter & Rothermund, 2002; Gollwitzer, 1999; Gollwitzer & Sheeran, 2006; Kato, 2012).

### The Concept of ‘Psychological Situation’

In this study, a situation is defined on the basis of theoretical ideas that differ from existing approaches to coping. In transactional theory, a situation is seen as a stressor, as “situation factors influencing appraisal” (Lazarus & Folkman, 1984, p. 82). Works based on this approach use the concepts of “stressor,” or “negative life event,” as the context in which an encounter takes place (Schwarzer & Schwarzer, 1996; Skinner, Edge, Altman, & Sherwood, 2003). An alternative view looks at a situation as a psychological phenomenon and focuses on subjective factors: perception and subjective experiencing *[perezhivanie]* of the situation.

According to the theory of psychological space, when people perceive and appraise life situations, they construct their own psychological space (Lewin, 1951). L.S. Vygotsky considered subjective experience to be “a unit for the study of personality and environment” (Vygotsky, 1984, p. 382). The process of experience combines objective circumstances and their perception by the person, which confers on these circumstances the status of a life event (Grishina, 2020).

In transactional theory, the subjective process involves appraisal of the stimulus as stressful (Primary Appraisal) and the subsequent determination of possible actions (Secondary Appraisal). These may be present or future events (Lazarus & Folkman, 1984; Peacock & Wong, 1990). The revised model adds that the appraisal stage may be followed by a stage of divergence between situational meaning and global aims and meanings, causing the experience of stress (Park & George, 2018). However, we believe that divergence between the situational and global meanings does not happen in all difficult life situations. Perhaps it may occur in one type of stressful (difficult) situation. On the whole, this approach assumes that there are some stress-generating characteristics of events which a person appraises in a certain way.

Our research has discovered a fact that cannot be explained by such theoretical notions. When asked to describe the content of an actual difficult life situation, 30% of respondents (n = 813) did not describe *the situation* as comprised of conditions, external circumstances, but as *internal experiences*^1^. Subjective factors play the key role here: self-determination, the search for one’s path in life, dilemmas, inner conflicts, lack of self-assurance, lack of will, “emotional dependence,” etc. The external context in this case is not decisive for appraisal and perception of a life difficulty, and a “negative life event” often does not actually take place in the respondent’s lifetime. Rather, many of these situations can be categorized as “tasks for meaning” (Leontiev, 1975), “the search for meaning” (Frankl, 1984), experiencing “the impossibility of realizing inner needs” (Vasilyuk, 2005); therefore, we prefer to see “the situation” as a psychological, subjective phenomenon. In accordance with activity theory, interaction with the world and the situation is effected through meaning-making (Leontiev, 1975; Sokolova, 2021). Hence the difficulty of the situation stems from how it is perceived.

In Russian psychology, the concept of a “difficult life situation” is used as an analogue of “negative life event” (Antsyferova, 1994); however, these concepts differ in some important ways. First, the concept of “a difficult life situation” does not carry an initial negative connotation (as distinct from “a negative life event”) and makes it possible to describe not only negative stressful experiences, but also the attractiveness of difficulty. Second, the concept of a “difficult life situation” makes it possible to study coping not only when resources are lacking (which leads to stress), but also when they are sufficient or even excessive (Bityutskaya, 2020).

### Theoretical Premises of This Study

The concept of “coping predictor” in this study is based on the following theoretical premises:

1. We proceed from the concept of the subjective or psychological situation as the space of the possible (Grishina, 2020; Heckhausen, 2008; Leontiev, 2011; Lewin, 1951). A difficult life situation is created not by the material structure of the situation and not even by its appraisal. A person, based on previous interaction with situations, “builds meanings” (Leontiev, 1975; Sokolova, 2021) and experiences them emotionally as a difficult life situation. The idea of interaction between an individual and a situation implies that the probable becomes real in the process of the subject’s activity. Accordingly, a difficult life situation may be explained as the space which simultaneously contains multiple stimuli: ways of fulfilling a task, the possibility of achieving a goal, obstacles, etc. By attaching significance to some stimuli and ignoring others, the person chooses between factors of the environment. Significant elements become the basis for “building an image” of the situation in individual consciousness. We use the concept “image of the world” to explain the mechanisms of this process (Leontiev, 1979). An actual image (including the image of a situation) can be formed when the subject sets into motion a process directed towards external stimuli (Smirnov, 2016). This process presupposes directed cognitive activity. These theoretical concepts formed the basis for creating our model of types of orientation in difficult situations.

2. By *orientation*, we mean the combination of cognitive, emotional, and motivational components conducive to certain coping efforts in a difficult life situation. Each orientation includes the following components: 1) types of stimuli that are considered significant; 2) representations of the world and of oneself upon which the subject relies when dealing with a difficult situation; 3) predictions; 4) emotions; 5) the difficulty of the goal. This concept describes the focus of attention on certain situational stimuli. While means of coping describe the person’s efforts, orientation points to the factors that determine a strategy for achieving (or not achieving) a difficult goal. The orientation model is designed to describe one type of difficult situation: life tasks. We identify *eight orientations:* five orientations characterize the focus of efforts on the approach to difficulties (drive, thoroughness, threat alert, opportunity orientation, obstacle orientation); and three orientations characterize avoidance of difficulties (flight or rejection, inaction, insouciance) (Bityutskaya, 2018). Our study looks at the version of life goal attainment that is accompanied by positive experiences in the coping process. This corresponds to the “drive” orientation, combined with reduced probability of a “rejection” orientation.

On the basis of qualitative research, we have described the orientation of being attracted to difficulties, “drive,” in which attaining a difficult goal generates pleasure from overcoming a difficulty, a sense of a rising flow of energy in anticipation of victory. Self-development is an important motive of drive, in which people are willing to exert themselves beyond what is required by the situation (Bityutskaya, 2018). The drive orientation in a difficult situation strongly correlates with proactive, problem-focused coping and positive reappraisal (Bityutskaya & Korneev, 2020a). In an interview, the respondents for whom drive is the preferred orientation said that in this state they have a sharpened sense of “being alive.” Analysis of the respondents’ subjective reports suggests that life satisfaction is at its highest when the person is aiming to overcome a difficult situation (Bityutskaya, 2018).

The orientation opposite to drive is orientation away from difficulties: “rejection.” This avoidance orientation in the face of a difficult task is explained by focusing on the loss of strength and on negative predictions and emotions, the futility of one’s efforts. In the absence of purposeful activity aimed at solving the task, the person focuses on attributing blame for the current state of affairs. This perception of the difficulty creates preconditions for avoidance (Bityutskaya, 2018). The indicators on that scale have a positive correlation with the coping strategies of wishful thinking (fantasizing) and self-blame, and a negative correlation with proactive coping, planning, and positive reappraisal (Bityutskaya & Korneev, 2020a).

3. In determining subjective appraisal, we proceed from appraisals in a context of categorization of the situation (Lazarus & Folkman, 1984). From the standpoint of activity theory (Leontiev, 1979), we define subjective appraisal as the process of image formation for a difficult life situation in the individual consciousness. The mechanism of appraisal is described in its relationship with the motive, the personal meaning in the situation, and the goal (Bityutskaya, 2013; Leontiev, 1975). Earlier we conducted a study aimed at revealing the basis upon which people categorize a situation as a difficult life situation. We identified eight criteria: the situation displays general features of a difficult life situation; does not lend itself to control; is unclear; demands a quick and active response; involves difficulty in making a decision; is difficult to predict; generates strong negative emotions; and is a threat to the future (Bityutskaya, 2013).

4. Proceeding from the ideas on appraising the difficulty of a situation and the orientation in the difficult situations model (Bityutskaya, 2013, 2018), we believe that in the process of interaction with the situation, the situation is first categorized and then appraised as being difficult on the basis of various criteria. Some appraisals of difficulty influence orientations whose function is to build up a readiness to use one way of coping or another. We do not rule out the possibility that appraisals may directly influence the actualization of coping methods.

5. In developing the structural models which were tested in this study, we were mindful of the two functions of coping described above: *approach* to the goal and *avoidance* of the goal. In this study, a negative correlation between approach and avoidance indicates a diminished tendency to avoid when experiencing attraction to the difficulty (and vice versa).

The aim of this study was to develop and test a path model that describes the influence of individual criteria of subjective appraisal and orientations in difficult situations to coping with planful problem solving, positive reappraisal, wishful thinking (fantasizing), and self-blame. In modeling, we took account of both direct and mediated relationships.

We tested the influence of subjective appraisal on difficult situations, and the effect on coping strategies of “drive” and “rejection” orientations in difficult situations. We believe that *subjective appraisal* of the difficulty of a situation (uncontrollability, unclearness, the intensity of negative emotions), as well as *orientations* in a difficult situation (drive or rejection), have an impact on the strength of (are predictors of) such *coping strategies* as planning, positive reappraisal, wishful thinking, and self-blame. Orientation in a difficult situation can play a mediating role between a subjective appraisal and coping strategies. The hypothesis of such connections is based on the model of orientations in difficult situations (Bityutskaya, 2018).

## Methods

### Participants

The sample consisted of 637 adult respondents aged between 19 and 64 (M_age_ = 24.2, SD = 6.25) who had given informed consent to take part in the study; 256 were males (M_age_ = 22.1; SD = 4.48) and 381 females (M_age_ = 25.6; SD = 6.84). They were students at Moscow universities in different specialties, as well as employees with a higher or secondary specialized education (bank employees, government officials, education and healthcare professionals, business managers) resident in Moscow and the Moscow Region. The sample size is sufficient for the path models presented below, as the ratio of sample to number of free parameters in the models is greater than 10:1, which is enough for obtaining reliable and unbiased estimates (Bentler & Chou, 1987).

### Assessment Procedure

In a questionnaire, we asked participants to describe a current life situation that involves solving a difficult task. After writing a description of the situation, participants responded to questions about their appraisals, orientations, and coping.

We received 637 descriptions of difficult situations across various spheres of life: professional and educational difficulties (implementing a major project, job-seeking, a tough exam, writing and defending a graduation paper/dissertation, combining work and study); material problems (buying an apartment, how to increase income); internal personal issues (self-identification, a life-changing choice, a dilemma, etc.); inter-personal relations; health problems.

### Measures

**Subjective appraisal** was measured by the questionnaire “Appraisal Criteria of the Difficulty of a Life Situation” (Bityutskaya, 2013). The questionnaire consists of 34 items by which the respondents assess the situation described on a scale from 0 (“no, totally wrong”) to 6 (“yes, absolutely right”). The results make it possible to identify why the respondent considers the situation as a difficult one. The questionnaire operationalizes subjective appraisals of a situation on the following scales, which correspond to criteria of difficulty: 1) *general features* of difficult situations; 2) *uncontrollability* of the situation; 3) *unclearness* (ambiguity) of the situation; 4) the need for a *quick and active response*; 5) *difficulty of making a decision* (dilemma); 6) *difficulty of predicting* the situation; 7) *negative emotions*; 8) *threat for the future.* The factor structure of the questionnaire was tested using confirmatory factor analysis on the material of difficult life situations of different contents (N = 736). The following fit indices were obtained: RMSEA = 0.044; CFI= 0.910; χ^2^ = 912.899, df = 378. This study used the results on scales 2, 3, 7.

**Orientations in a difficult situation** were measured by the situational version of the questionnaire “Types of Orientations in Difficult Situation” (TODS; Bityutskaya & Korneev, 2020b). The questionnaire comprises 65 items which respondents must answer relative to the situation described and assess on a scale from 0 to 3 (0 — “totally wrong,” 1 — “somewhat wrong”; 2 — “somewhat right,” and 3 — “absolutely right”). The questionnaire is designed to diagnose the perception of a difficult situation in the respondent’s life as described by the respondent. TODS differentiates the two poles of the coping dimension “approach–avoidance,” by introducing orientations that describe the perception of the situation as a complex of cognitive, emotional, and motivational components (goal level, predicting, emotions, focus of efforts, etc.). The questionnaire makes it possible to diagnose eight orientations: five orientations characterize the subject’s efforts to approach difficulties (*drive, thoroughness, threat alert, opportunity orientation, obstacle orientation*); three orientations involve avoiding difficulties (*rejection, inaction, insouciance*). *Drive* describes an attitude that welcomes a difficult situation and is accompanied by a surge of enthusiasm and positive emotions. *Rejection* characterizes the perception of a difficult situation as a waste of time and energy and a source of anxiety. The questionnaire’s structural model was evaluated in studying different difficult situations of 687 adults. The model fits well: RMSEA = 0.049, CFI= 0.900, χ^2^ = 3,068.835, df = 1,171.

**Ways of coping** were assessed with the Russian version of the “Ways of Coping Checklist” (revised). The WCC consists of 66 items that the study participants evaluate on a 4-point ordinal response scale including 0 (“not used”), 1 (“used somewhat”), 2 (“used quite a bit”), and 3 (“used a great deal”). The questionnaire was adapted for studies of Russian-language samples (N = 727) in order to study coping with a situation that the respondent considers to be difficult and urgent. The questionnaire structure was developed on the basis of material concerning various difficulties, using exploratory and confirmatory factor analysis as well as expert evaluations. We selected nine factors that were used in the original versions of the Checklist (Folkman & Lazarus, 1985; Folkman, Lazarus, Dunkel-Schetter, DeLongis, & Gruen, 1986), but were not identical to the original versions in terms of the scales. These factors are as follows: 1) *planful problem-solving*, deliberate problem-focused efforts to alter the situation, coupled with an analytic approach to solving the problem; 2) *seeking social support*; 3) *positive reappraisal*, efforts to create positive meaning by focusing on personal growth; 4) *confrontive coping*; 5) *self-controlling*; 6) *self-blame*, acknowledging one’s own role in the problem with a concomitant theme of reproach and trying to put things right; 7) *wishful thinking (fantasizing)* and hope for a miracle; 8) *distancing*; 9) *escape-avoidance.* The factor model of the Russian-language version of the WCC has the following estimates: RMSEA = 0.047; CFI= 0.89; χ^2^ = 2,634; df = 1, 011 (Bityutskaya, 2014). In this study we used the results of scales 1 (Cronbach’s α = 0.712 in our sample), 3 (α = 0.817), 6 (α = 0.521), and 7 (α = 0.736).

We employed the following strategies to rule out common method bias (Jordan & Troth, 2019): the participants analyzed a difficult situation that they had actually experienced subjectively, which minimizes retrospective distortions and makes it possible to obtain data on the subjective situation. Some of the scales involve elements with reverse scaling. Finally, we motivated the respondents to give honest answers by promising (and keeping the promise) to give feedback in the form of an individual profile of appraisal and coping with the difficult life situation.

### Statistical Analysis

The path models were assessed in an Mplus package, version 8.3. The analysis used the method of maximum likelihood estimation with robust standard errors, calculated by using a sandwich estimator (estimator MLR in Mplus). To assess the distribution of the variables included in the model, a descriptive statistic was calculated and the normality of the distribution was assessed by the Shapiro-Wilk test.

## Results

### Descriptive Statistics and Correlation Analysis

The descriptive statistics of the scales and results of testing of their normality are presented in *Table 1.*

**Table 1:** Descriptive statistics of the scales

Descriptive statistics	Uncontrollability	Unclearness	Negative emotions	Drive	Rejection	Problem–solving	Reappraisal	Wishful thinking	Self–blame
Mean	1.983	2.348	3.424	1.757	1.428	1.838	1.824	1.352	1.427
Standard deviation	1.201	1.113	1.316	0.628	0.598	0.587	0.707	0.688	0.672
Skewness	0.600	0.171	–0.124	–0.231	0.191	–0.135	–0.289	0.165	0.107
Std. error skewness	0.097	0.097	0.097	0.097	0.097	0.097	0.097	0.097	0.097
Shapiro-Wilk W	0.966^*^	0.991^*^	0.982^*^	0.987^*^	0.990^*^	0.988^*^	0.976^*^	0.984^*^	0.981^*^

Note. ^*^ p <0.001

It will be seen from the table that all the variables have a distribution different from the normal, which is due to a pronounced skewness. Because of this, the use of a robust estimates in confirmatory factor analysis is adequate. The Pearson correlations between the variables are presented in *Table 2.*

**Table 2:** Pearson correlation coefficients between the scales

Scale	Uncontrollability	Unclearness	Negative emotions	Drive	Rejection	Problem–solving	Reappraisal	Wishful thinking
Unclearness	0.529^***^	—						
Negative emotions	0.446^***^	0.431^***^	—					
Drive	–0.414^***^	–0.236^***^	–0.341^***^	—				
Rejection	0.464^***^	0.504^***^	0.616^***^	–0.454^***^	—			
Problem-solving	–0.346^***^	–0.293^***^	–0.225^***^	0.493^***^	–0.333^***^	—		
Reappraisal	–0.265^***^	–0.117^**^	–0.171^***^	0.640^***^	–0.293^***^	0.573^***^	—	
Wishful thinking	0.334^***^	0.302^***^	0.337^***^	–0.114^**^	0.388^***^	–0.071	0.024	—
Self-blame	0.082^*^	0.209^***^	0.333^***^	–0.031^***^	0.355^***^	0.020	0.081^*^	0.345^***^

Note. ^*^ p < 0.05, ^**^ p < 0.01, ^***^ p < 0.001

Analysis in most cases revealed high correlations between the variables. This indicates that the constructs included in the analysis are interconnected; however, the structure of pair correlations is difficult to describe through simple analysis. We will therefore describe in more detail the path model, based on theoretical suppositions concerning the mechanisms of the impact of subjective appraisal indicators on orientations in difficult situations, which in turn influence the dominance of certain ways of coping.

### Path Analysis

The first path model included three indicators of the subjective appraisal scale as independent variables: *uncontrollability, unclearness*, and *negative emotions*, as well as two orientations in difficult situations: *drive* and *rejection.* The dependent variables were ways of coping: *planful problem-solving*, *positive reappraisal*, *wishful thinking*, and *self-blame.*

*Planful problem-solving* and *positive reappraisal*, according to the model, are impacted by *negative emotions* and *drive*; and *wishful thinking* and *self-blame* by *uncontrollability*, *unclearness*, *negative emotions*, and *rejection.* A correlation is allowed between errors of pairs of dependent variables: *planful problem-solving*–*positive reappraisal* and *wishful thinking*–*self-blame.*

**Figure 1.**
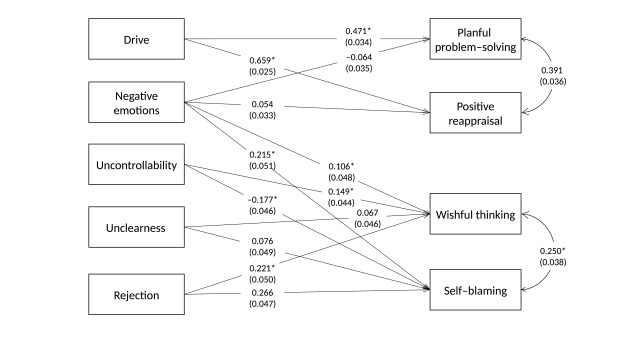
Subjective appraisals and orientations in difficult situations as predictors of coping strategies: direct influence model, Model 1.

The fit indices of the model turned out to be fairly good: χ^2^(12) = 74.882, SRMR = 0.061, RMSEA = 0.091 (with 95% confidence interval [0.072; 0.111]), CFI= 0.930, TLI = 0.848. Some estimates, though, turned out to be close to zero. The standardized coefficients are shown in *Figure 1*; the full table of coefficients with confidence intervals is in Appendix A (*Table A1*).

The results reveal low estimates of the impact of *negative emotions* on *planful problem-solving* and *positive reappraisal*, and of *unclearness* on *wishful thinking* and *self-blame.*

Proceeding from the results obtained, and from a theoretical analysis of the relationship between the variables included in the analysis, an alternative (second) model was built, in which some direct links between independent and dependent variables were replaced or complemented by mediated ones. This applies to links between a subjective appraisal and the “ways of coping” indicators. We assumed that some of these links may be mediated by orientations in difficult situations. That is, a subjective appraisal does not merely affect the prevalence of this or that strategy directly, but also (and in some cases only) the orientation in a difficult situation, which in turn may prompt a particular way of coping.

The second path model included *uncontrollability, unclearness*, and *negative emotions* as primary independent variables. All these variables, according to the second model, 1) may influence the *rejection* orientation; 2) *uncontrollability* and *negative emotions* influence the *drive* orientation; and 3) *uncontrollability* directly impacts *wishful thinking* and *self-blame*, and *negative emotions* impact *self-blame.*

In turn, two orientations in difficult situations impact ways of coping in the following manner: *drive* has a positive impact on *planful problem-solving* and *positive reappraisal*, and *rejection* has a meaningful positive impact on *planful problem-solving*, *wishful thinking*, and *self-blame.*

The model also includes correlations among all three subjective appraisal scales, between *drive* and *rejection*, and also between residuals of the two pairs of independent variables: *planful problem-solving*–*positive reappraisal*, and *wishful thinking*–*self-blame*

**Figure 2.**
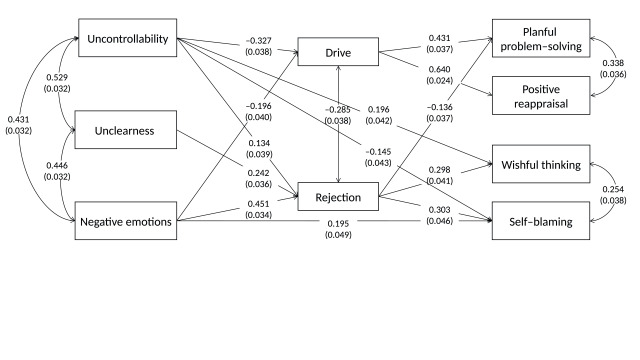
Subjective appraisals and orientations in di# cult situations as predictors of coping strategies: mediated in" uence model, Model 2.

The fit indices of the second model proved to be better than those of the first: χ^2^(17) = 79.025, SRMR = 0.051, RMSEA = 0.076 (with 95% confidence interval [0.059; 0.093]), CFI= 0.956, TLI = 0.915. Because the two models considered above differ in structure and are not nested, a direct comparison is impossible, but the fit indices show that the second model agrees with the data better than the first. The standardized coefficients are shown in *Figure 2*, and the full table of coefficients with confidence intervals is in Appendix 1 (*Table A2*).

The results of analysis show a significant negative influence of *uncontrollability* on *drive* and a positive influence on *rejection.* Similarly, *uncontrollability* has a significant positive influence on *wishful thinking* and *self-blame.* *Unclearness* has a positive influence on *rejection*, and *negative emotions* have a positive influence on *rejection* and a weaker, but still significant negative influence on *drive.* Furthermore, *drive* exerts a positive influence on *planful problem-solving* and *positive reappraisal*, and *rejection* has a significant positive influence on *wishful thinking* and *self*-*blame.*

Moreover, according to the model, all three subjective appraisal indicators significantly correlate with one another. Likewise, the pairs of coping methods—*planful problem-solving*–*positive reappraisal*, and *wishful thinking*–*self-blame*—have significant positive correlations. Finally, a negative correlation has been obtained between the drive and rejection orientations included in the model.

## Discussion

Previous studies of coping predictors mainly proceeded from the transactional theory of stress and coping (Lazarus & Folkman, 1984). In contrast, this study uses an approach in which coping predictors are components of the psychological (perceived) situation. We have considered coping as a process that permits one to achieve a difficult life goal. The participant does not only appraise the difficult task, but is a subject who vigorously interacts with the situation through meaning-making (Leontiev, 1975; Sokolova, 2021). We were verifying a hypothesis on the combined influence on coping strategies of appraisals and orientations in difficult situations. We did not check whether the person felt stress in a life situation, but the instruction asked the respondents to describe a real life task that needed to be solved and then to analyze their experiences, emotions, appraisals, and predictions. We reconstructed the perceived (psychological) situation on that basis.

This study affords a description of perceived situation characteristics as predictors of coping strategies. We distinguish between coping strategies themselves and predictors of coping: cognitive appraisals of a situation, orientations as a complex of perceptual characteristics. In this context, the idea that approach–avoidance is cognitive activity is more suitable for us. Orientation in a difficult situation is largely related to the orientation of attention (Roth & Cohen, 1986; Skinner et al., 2003).

An evaluation of the two models has proved that it is possible to describe the range of predictors of coping methods through a simple model containing only the direct effects of indicators of subjective appraisal and orientations in difficult situations. Some direct effects, however, turned out to be insignificant.

The second model includes not only direct but also mediated links between independent and dependent variables. For instance, subjective appraisal indicators have an impact on orientations in difficult situations, which in turn affect the methods of coping. This model has demonstrated better data fit, showing that the structure of coping strategy predictors can be more complex than the direct influence of subjective appraisals and orientations on one way of coping or another. Thus, for example, in the first model, direct correlations between *negative emotions* and *planful problem-solving* turned out to be insignificant. But according to the second model, *negative emotions* may have a significant (negative) impact on *drive*, which in turn significantly influences *planful problem-solving.* In other words, the less intense are the *negative emotions*, the more manifest is *drive*, which makes *planful problem-solving* more likely. This indicates that direct and mediated influences may differ not only in intensity, but also in the direction (positive or negative) of impact.

Thus, the mediated influence model allows a systematic description of one of the orientations of being attracted to difficulties that make people feel at their most active, focused on attaining the goal and experiencing feelings of pleasure and anticipation of success (drive). This is congruent with the definition of a challenge from the transactional theory of stress and coping (Lazarus & Folkman, 1984). However, there are differences: “drive” is not confined to an evaluation, but is an entire “pattern” which includes a sense that a situation is manageable; positive emotions (interest, curiosity, and inspiration); positive predictions concerning the development of events; and increased complexity of the task. Such people feel bored when confronted with tasks of a low degree of difficulty. This description is similar to the characteristic of intrinsically motivated, or autotelic, activity: “activity rewarding in and of itself…, quite apart from its end product or any extrinsic good that might result from the activity” (Nakamura & Csikszentmihalyi, 2002). It is also possible to compare drive orientation with “oversituational activity,” which is characterized by the growth and development of human capabilities (Petrovsky, 2010).

We have demonstrated the important role of subjective control and lowering of negative emotions for experiencing drive. Drive itself is a predictor of the actualization of planful coping and a positive reappraisal of the situation. While the majority of modern approaches see coping strategies as a predictor of psychological well-being, we have described the experience of drive, which implies a sense of the fullness of living not as a result of, but in the process of, coping with a difficult situation. Herein lies the originality of the proposed model.

Proceeding from the idea that goal attainment is connected with resistance to distractive stimuli (Brandstädter & Rothermund, 2002; Gollwitzer & Sheeran, 2006), we have introduced the factor of wishful thinking (or fantasizing), which is connected with self-blame. Our results show that these coping strategies are actualized when a difficult situation is perceived as a waste of time and effort, with an anxious focus on negative predictions of how the situation will develop (rejection). The most powerful predictor of rejection is negative emotions generated by attaining a difficult goal, as well as the less strong but still significant predictors of uncontrollability and unclearness of a situation (the lack of a clear vision or understanding of how to act in order to achieve the desired result).

Our results are comparable to the theoretical conceptions and empirical evidence on perceived control, which influences how well people act and plan, or remain passive and avoidant (Skinner, 1995). Our study of subjective appraisals of life situations corroborates the results of studies of control as power. Power is associated with the clarity of focusing on the goal, and also encourages work on the achievement of goals (Guinote, 2017; Scherer & Moors, 2019).

According to our data, subjective control is not only a predictor of orientations, but also directly influences the actualization of wishful thinking and self-blame. Interestingly, with increasing subjective control, self-blame increases and avoidance decreases.

The connections revealed in our study correspond to the conceptual model of orientations in difficult situations and demonstrates their mediatory role.

## Conclusion

The purpose of this study was to develop and test two structural models in which subjective appraisals and orientations in difficult situations were considered as predictors of coping strategies (*planful problem-solving*, *positive reappraisal*, *wishful thinking*, and *self-blame*). The first model assumes a direct influence of coping predictors (subjective appraisals: *uncontrollability*, *unclearness*, *negative emotions*; and orientations: *drive* and *rejection*). In the second model, orientations in difficult situations are constructs mediating the connection between subjective appraisals and coping strategies. Comparison of the two models shows that the more complex second model fits better with the data than the first model.

The proposed models describe two opposite strategies. The first strategy implies the achievement of a difficult goal with positive emotions, a feeling of satisfaction with life in the process of overcoming difficulties (drive). The second is associated with an orientation toward avoiding difficulties, minimizing the waste of effort and resources (rejection).

The results can be interpreted as corroboration of the idea of interaction between the subject and the situation. The process of such interaction involves appraisal of difficulty, which influences orientation in difficult situations. In turn, orientations are predictors of coping strategies. The characteristics of the psychological situation determine coping, which may be oriented either toward approach to or avoidance of the goal.

## Limitations

The study has some limitations. First, this is a cross-sectional survey. We plan to further test the identified relationships using an experimental design.

Second, we considered only one type of situation—“tasks”—involving the achievement of a difficult goal, but we were presented with various situations in different spheres of life (material, professional challenges, etc.). Subsequently, it would be interesting to answer the question: Is coping different for different types of situation (situations)? Or are the connections we have found sufficiently universal that they manifest themselves in situations or “tasks” belonging to different domains of life?

Third, we looked at two aspects of achieving a difficult goal, related to approaching and avoiding. Important aspects have been left out of attention: mechanisms that facilitate the adjustment of goals and the concomitant ability to abandon an ineffective coping strategy (Kato, 2012). We believe that in considering coping as a dynamic process, these are stages of the process of appraisal and coping that could be most effectively studied in research designed to model the process of difficult goal attainment and to combine observation and experiment.

## Footnotes

1 The same proportion (30% of the situations described by respondents) was found in our study where people described their difficult life tasks.

## Appendix A

**Table A1:** Standardized path coefficient for Model 1

Terms*	Estimate	Standard error	95% confidence interval
Drive on Planful problem-solving	0.471	0.034	[0.404; 0.538]
Negative emotions on Planful problem-solving	–0.064	0.035	[–0.133; 0.005]
Drive on Positive reappraisal	0.659	0.025	[0.610; 0.708]
Negative emotions on Positive reappraisal	0.054	0.033	[–0.011; 0.119]
Rejection on Self-blame	0.266	0.047	[0.174; 0.358]
Unclearness on Self-blame	–0.177	0.046	[–0.267; –0.087]
Uncontrollability on Self-blame	0.076	0.049	[–0.020; 0.172]
Negative emotions on Self-blame	0.215	0.051	[0.115; 0.315]
Rejection on Wishful thinking	0.221	0.05	[0.123; 0.319]
Unclearness on Wishful thinking	0.149	0.044	[0.063; 0.235]
Uncontrollability on Wishful thinking	0.067	0.046	[–0.023; 0.157]
Negative emotions on Wishful thinking	0.106	0.048	[0.012; 0.200]
Wishful thinking with Self-blame	0.25	0.038	[0.175; 0.325]
Positive reappraisal with Planful problem-solving	0.391	0.036	[0.320; 0.462]

*Note. ^*^ “on” – regression; “with” – correlation*

**Table A2:** Standardized path coefficient for Model 2

Terms*	Estimate	Standard error	95% confidence interval
Drive on Planful problem-solving	0.431	0.037	[0.358; 0.504]
Rejection on Planful problem-solving	–0.136	0.037	[–0.209; –0.063]
Drive on Positive reappraisal	0.64	0.024	[0.593; 0.687]
Rejection on Self-blaming	0.303	0.046	[0.213; 0.393]
Negative emotions on Self-blaming	0.195	0.049	[0.099; 0.291]
Uncontrollability on Self-blaming	–0.145	0.043	[–0.229; –0.061]
Rejection on Wishful thinking	0.298	0.041	[0.218; 0.378]
Uncontrollability on Wishful thinking	0.196	0.042	[0.114; 0.278]
Uncontrollability on Drive	–0.327	0.038	[–0.401; –0.253]
Negative emotions on Drive	–0.196	0.04	[–0.274; –0.118]
Uncontrollability on Rejection	0.134	0.039	[0.058; 0.210]
Unclearness on Rejection	0.242	0.036	[0.171; 0.313]
Negative emotions on Rejection	0.451	0.034	[0.384; 0.518]
Rejection with Drive	–0.285	0.038	[–0.359; –0.211]
Unclearness with Uncontrollability	0.529	0.032	[0.466; 0.592]
Negative emotions with Uncontrollability	0.446	0.032	[0.383; 0.509]
Negative emotions with Unclearness	0.431	0.033	[0.366; 0.496]
Wishful thinking with Self-blaming	0.254	0.038	[0.180; 0.328]
Planful problem-solving with positive reappraisal	0.388	0.036	[0.317; 0.459]

*Note. ^*^ “on” – regression; “with” – correlation*
